# Notes and News

**Published:** 1892-09-10

**Authors:** 


### Notes and News.

Collected and Communicated.

It cannot be doubted that the hope of better living elsewhere has been one of the deterrent causes in the path of enlisting into the army. The quantity and quality of the men's rations is now being generally improved, and this should tend to increase the percentage of well-grown and superior amongst the recipients of the proverbial "shilling."

It is said that the Vestries having control of the Regent's Canal, in view of the possible cholera outbreak, propose to commence proceedings to render it more sanitary. We cannot foresee any possible method of effecting this without a preliminary disturbance of the canal bed, which at this time of the year would surely be unbearable for the luckless dwellers in the vicinity, and more likely to produce typhoid than prevent cholera.

Every day sees the bicycle more popular. As a convenient and healthy mode of travelling it is almost unrivalled, but whilst its presence is becoming so frequent, both in town and country, we regret to perceive a growing carelessness on the part of cyclists, which amounts to actual danger to the less active of pedestrians. Quite recently a lady, only just convalescent from a long illness, whilst walking along a country road, was knocked down by a passing cyclist, and so injured, that her recovery is doubtful. The rider's carelessness has been duly punished, but this can but afford small consolation to the lady's friends.

We have always contended that an education which does not develop the practical common-sense and intelligence is really no education at all. And for attention to that branch of the School Board curriculum entitled "domestic economy," we have made a special plea. The absurd answers given in a recent number of *The Journal of Education* on this subject, as elicited from a class of big girls, should serve to show the superficial knowledge of common things which is only too general in schools. One girl states that "Crétins are generally served in green pea-soup," whilst another statement is that "The body wastes away by the continual working of the bones together, and as the process goes on every day, the bones get thinner and smaller."

Mr. Robert Marcus Gunn, M.A., F.R.C.S., has accepted appointment as Hon. Ophthalmic Surgeon to the Cheyne Hospital for Sick and Incurable Children, Cheyne Walk, Chelsea. The Committee have recently received a donation of 100 guineas to the funds of the hospital from Mr. William White, of Chelsea.

We are glad to see that attention is again being called to the nuisance of street music. The comparative absence of "light-heartedness" in the English nature, is very generally put down to absence of sun in our climate. In our towns it would be difficult to exaggerate the extent to which the gloom of a bad day is aggravated by the depressing groans of barrel organs, concertinas, and other instruments of torture; what health, or what spirits, could bear up under such conditions?

It appears that the Prussian Medical Board are drawing up a Bill for the prevention of epidemics, which is to be submitted to legislature at an early date. When the contents are published we may learn a wrinkle or two ourselves; but what lessons can be of real value to us, if we, dwellers on an island, wantonly throw away our natural defences against the introduction of disease by permitting travellers from infected districts, under any circumstances, to land on our shores without at least a week's quarantine on board ship?

The questionable policy of deferring necessary repairs to buildings to an indefinite date is fully exemplified in the present position of the Royal South Hants Infirmary. It appears that the building has been allowed to fall into such a state of disrepair that a large sum of money will be required speedily to do the necessary work. The public will no doubt be asked to find the money for the undertaking, but might not such calls upon the public purse be avoided by a more far seeing, and certainly, in the long run, less expensive policy? Had the building been kept in repair from year to year there seems no reason to suppose that the funds of the Institution could have borne the cost which, when falling in one year, they cannot do without trenching on their capital. The Committee correctly consider the case to be one of extreme emergency, but are not the subscribers justified in asking why the emergency should have been allowed to arise?

Each year brings its list of victims to Alpine adventure. We cannot regret that spirit of emulation and enterprise which causes the larger number of losses to be amongst our own countrymen, but we must all feel the deepest sympathy with the friends of those who are lost through a want of the most ordinary of prudence. Alpine climbers will all agree that Mr. Nettleship would not have lost his life had the guides accompanying him exercised the common sense which should be expected from men experienced in the weather of the Alps. Ourselves, we cannot feel quite satisfied with the account of the mishap as given by the guides. So sudden a cessation of life seems very strange in a man of Mr. Nettleship's hardy constitution. But after all, the fact, not the manner of his death, concerns us most. The loss to Oxford, and to Baliol College in particular, is very great. Mr. Nettleship has been described as "the pivot on which Baliol turned," although through his unassuming modesty he habitually avoided occupying a prominent position.

The adjourned meeting of the Addenbrooke's Hospital, Cambridge, has been held, with the result that the proposed drainage scheme is to be carried out. This will necessitate the entire closing of the hospital for a time. A scheme for temporary accommodation is to be considered later.

Is it, we wonder, a distrust in the powers that be, at the Oxford Infirmary, to cope with the requirements of the times, or for the purpose of setting them a good example, that the City authorities of Oxford are taking such complete and admirable measures to insure the best sanitary condition of streets and dwellings, in view of a possible cholera outbreak? Whatever reasons may have influenced the authorities, the outcome is to their credit, and their methodical proceedings might with advantage be adopted by all other towns in the kingdom.

The new isolation block, in connection with the Sunderland Infirmary, was opened on Monday last. In conjunction with it are mortuary and post-mortem room. Externally the block is of handsome appearance, whilst internally it is exceedingly complete. The accommodation consists of two wards, with annexes. Each ward has its bath-room fitted with Rufford's baths. The rooms are tiled. There are closets appertaining to each ward, provided, however, with well-ventilated space between ward and closet. The ward walls are painted with Silica paint. The nurses' rooms join the wards, and command a view of the interior. All sinks and fittings are carefully chosen.

Despite the strenuous and continued opposition made by the inhabitants at Tottenham, the erection of the fever hospital within their midst is being pushed to the utmost. Two contractors have the work in hand, and are employing so large a staff of workmen that, if the building continues uninterrupted, all will be completed in a month. Some delay, however, is threatened, through a strike amongst the joiners for a slight increase of wages. The completion of the hospital is looked forward to by the medical authorities with impatience, as the fever epidemic in the metropolis and suburbs shows no decrease, and all existing accommodation is taxed to the utmost.

The necessary preparations in view of a possible visitation of cholera, are causing many corporations anxiety and difficulty. It is necessary that accommodation should be ready for the reception of patients, and in the event of no fever hospital existing, some other building has to be fixed upon. The Corporation of Sunderland have experienced all the difficulties of the situation, and so strong has the opposition to the use of the building of their choice from the inhabitants of Monkwearmouth proved, that Mayor and Corporation, accompanied by the engineer of the River Wear, are to make a journey of discovery up the river in order to find a suitable mooring for a floating hospital. We can quite understand that a floating site is infinitely preferable where it can be obtained, but there are many authorities who will be sorely troubled to meet both the objections to utilising existing accommodation and the call on the ratepayers' pocket where new premises are demanded.

The American census bulletin on homicide gives us some interesting facts. Throughout the American prisons, of the eighty thousand inmates, over seven thousand are charged with homicide. Amongst the white criminals 3,200 were native born, and 1,200 were of foreign birth. Amongst the foreigners the Irish take the lead as to numbers, the number of Irish emigrants to America is only exceeded by the German. Nowadays, when a large number of people are inclined to look upon the education of the lower classes as pernicious, it is worthy of note that more than four-fifths of these American homicides had no trade, and eighty-eight per cent. of them could neither read nor write. Some of the States have abolished capital punishment, and in these it is noted that the number of homicides is rather less than in those where it is still enforced.

